# Off the beaten pathway: Powering carbon capture with alternative photosynthetic electron transfer pathways

**DOI:** 10.1093/plcell/koae155

**Published:** 2024-05-24

**Authors:** Guy Levin

**Affiliations:** Assistant Features Editor, The Plant Cell, American Society of Plant Biologists; Faculty of Biology, Technion, Haifa, 32000, Israel

During photosynthesis, light drives the formation of NADPH by powering a linear electron flow (LEF) from water, through PSII and I, to NADP. Additionally, LEF generates a proton motive force (*pmf*) across the thylakoid membrane, which is utilized to generate ATP. However, LEF is insufficient for providing the ATP requirements for various metabolic processes, including carbon fixation in the Calvin-Benson-Bassham cycle. Alternative electron pathways may provide the additional required energy (Allen 2003). During cyclic electron flow (CEF), electrons are recycled around PSI (Nawrocki et al. 2019). In pseudo-CEF (PCEF), electrons are ejected from the photosynthetic electron chain by reducing O_2_ on the PSI acceptor side (Chaux et al. 2017). Both pathways promote *pmf* generation and ATP production (Fig. 1). Transferring reductants from the chloroplast to the mitochondria, where they are converted to ATP in the respiratory chain and shuttled back to the chloroplast, may also provide additional ATP for carbon fixation (Raghavendra and Padmasree 2003). This process is termed chloroplast to mitochondria electron flow (CMEF; Fig. 1). Although the mechanisms of alternative photosynthetic electron flows were described, the importance of each pathway for carbon fixation remains elusive.

**Figure 1.**
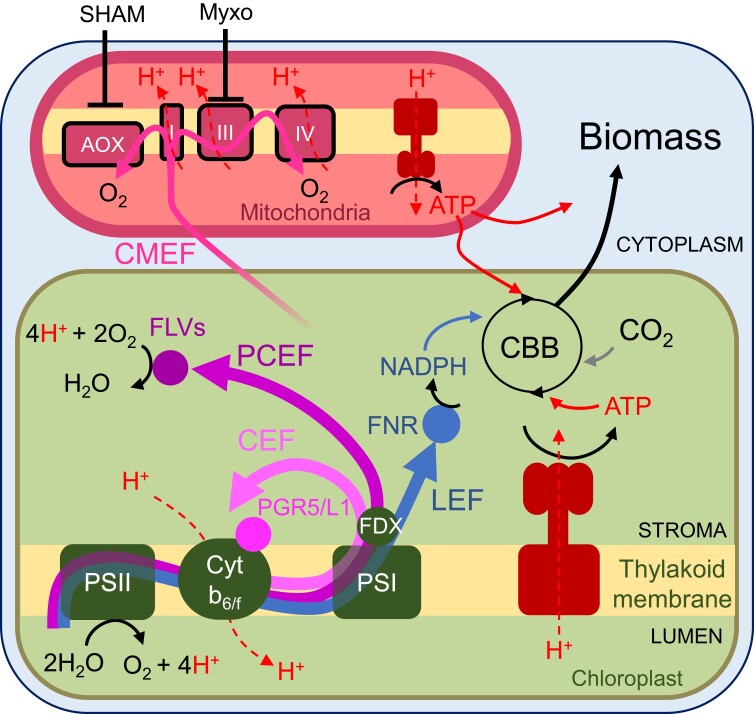
Electron transport pathways operating during oxygenic photosynthesis. LEF, CEF, and PCEF promote the formation of a *pmf* force by translocating protons across the thylakoid membrane. *pmf* drives ATP synthesis. In CMEF, reductants from the chloroplast promote *pmf* and ATP synthesis in the mitochondria via the respiratory chain. ATP from all pathways can energize carbon fixation, with CMEF being the most efficient. CBB, Calvin Benson Bassham cycle; FDX, ferredoxin; FLVs, flavodiiron proteins; FNR, ferredoxin-NADP + reductase; Myxo, myxothiazol; PGR5/L1, PROTON GRADIENT REGULATION 5/like-1; SHAM, salicyl hydroxamic acid; I, III, IV, respiratory complexes I, III, and IV. Reprinted from Peltier et al. (2024), Figure 1.

Following the discovery by Burlacot et al. (2022) that CEF, PCEF, and CMEF provide energy for the carbon concentrating mechanism, in this issue, **Gilles Peltier and colleagues (Peltier et al. 2024)** quantify the contribution, extent, and efficiency of each pathway by measuring their capacity to sustain net photosynthesis and CO_2_ fixation in the microalga Chlamydomonas (*C. reinhardtii*). The authors measured the O_2_ exchange rates in Chlamydomonas mutants, which are impaired in both CEF and PCEF, and showed that they are capable of maintaining near-maximal net photosynthesis, suggesting that CMEF compensates for the absence of CEF and PCEF. However, if respiration (CMEF) is additionally inhibited, photosynthesis is completely abolished. In wild-type cells, inhibiting respiration leads to a ∼15% decrease in net photosynthesis, suggesting that CEF and PCEF can mostly compensate for the loss of CMEF. Inhibiting respiration in Chlamydomonas mutated in either *pgrl1* (CEF) or *flvB* (PCEF) showed that each pathway may energize 60% of net photosynthesis. Together, these results show that all 3 pathways are the predominant ATP producers for the cell.

Similar results were obtained when measuring inorganic carbon uptake, suggesting that CEF, PCEF, and CMEF can separately power carbon fixation; however, only CMEF, and thus respiration, sustains nearly maximal net photosynthesis in the absence of the other 2 pathways. CMEF provides ATP for carbon fixation via 2 pathways involving complex III-IV or alternative oxidase (AOX) in the mitochondria (Fig. 1). Blocking each of the pathways in the CEF-PCEF double mutants revealed that the complex III-IV pathway can energize most of the net photosynthesis, while the AOX pathway sustains ∼45%. To determine the efficiency of PCEF and CMEF, the authors introduce an equation to calculate a metric they termed net e^−^ yield, representing the capacity of electrons in a specific pathway to facilitate net photosynthesis, thus reflecting ATP synthesis used for metabolic purposes. The net e^−^ yields of PCEF and CMEF were 0.65 and 0.85, respectively. In CEF, which is impractical to measure under physiologically relevant conditions (Burlacot et al. 2022), the net e^−^ yield is estimated at 0.55. These results highlight CMEF as the most electron-efficient pathway for energizing carbon fixation, having the highest net e^−^ yield. Finally, the authors measured the contribution of each pathway to powering net photosynthesis during steady-state photosynthesis and concluded that CMEF sustains 30% to 60% of CO_2_ fixation, PCEF 10% to 30%, and CEF 25% to 30%.

In summary, the authors reveal that CEF, PCEF, and 2 CMEF pathways can each partially sustain carbon fixation and compensate for the absence of the others, with CMEF being the most efficient and only pathway able to sustain almost maximal net photosynthesis (Fig. 1). These results suggest that CMEF is vital for carbon capture during photosynthesis and provide a basis for planning strategies to enhance carbon fixation in photosynthetic organisms.

## Dive Curated Terms

The following phenotypic, genotypic, and functional terms are of significance to the work described in this paper:

ATP CHEBI: CHEBI:15422

NADP CHEBI: CHEBI:25523

NADPH CHEBI: CHEBI:16474

electron CHEBI: CHEBI:10545

FLVB Gramene: Cre16.g691800
FLVB Araport: Cre16.g691800

PGRL1 Gramene: Cre07.g340200
PGRL1 Araport: Cre07.g340200

## References

[koae155-B1] Allen JF . Cyclic, pseudocyclic and noncyclic photophosphorylation: new links in the chain. Trends Plant Sci. 2003:8(1):15–19. 10.1016/S1360-1385(02)00006-7 12523995

[koae155-B2] Burlacot A , Dao O, Auroy P, Cuiné S, Li-Beisson Y, Peltier G. Alternative photosynthesis pathways drive the algal CO2-concentrating mechanism. Nature. 2022:605(7909):366–371. 10.1038/s41586-022-04662-9 35477755

[koae155-B3] Chaux F , Burlacot A, Mekhalfi M, Auroy P, Blangy S, Richaud P, Peltier G. Flavodiiron proteins promote fast and transient O_2_ photoreduction in *Chlamydomonas*. Plant Physiol. 2017:174(3):1825–1836. 10.1104/pp.17.00421 28487478 PMC5490913

[koae155-B4] Nawrocki WJ , Bailleul B, Picot D, Cardol P, Rappaport F, Wollman F-A, Joliot P. The mechanism of cyclic electron flow. Biochim Biophys Acta (BBA)—Bioenerget. 2019:1860(5):433–438. 10.1016/j.bbabio.2018.12.005 30827891

[koae155-B5] Peltier G , Stoffel C, Findinier J, Madireddi SK, Dao O, Epting V, Morin A, Grossman A, Li-Beisson Y, Burlacot A. Alternative electron pathways of photosynthesis power green algal CO_2_ capture. Plant Cell. 2024:36(10):4132–4142. 10.1093/plcell/koae143 PMC11449004 38739547

[koae155-B6] Raghavendra AS , Padmasree K. Beneficial interactions of mitochondrial metabolism with photosynthetic carbon assimilation. Trends Plant Sci. 2003:8(11):546–553. 10.1016/j.tplants.2003.09.015. 14607100

